# Longitudinal Follow Up of Immune Responses to SARS-CoV-2 in Health Care Workers in Sweden With Several Different Commercial IgG-Assays, Measurement of Neutralizing Antibodies and CD4^+^ T-Cell Responses

**DOI:** 10.3389/fimmu.2021.750448

**Published:** 2021-11-02

**Authors:** Emelie Marklund, Susannah Leach, Kristina Nyström, Anna Lundgren, Jan-Åke Liljeqvist, Staffan Nilsson, Aylin Yilmaz, Lars-Magnus Andersson, Mats Bemark, Magnus Gisslén

**Affiliations:** ^1^ Department of Infectious Diseases, Institute of Biomedicine, Sahlgrenska Academy, University of Gothenburg, Gothenburg, Sweden; ^2^ Department of Infectious Diseases, Sahlgrenska University Hospital, Gothenburg, Sweden; ^3^ Department of Microbiology and Immunology, Institute of Biomedicine, Sahlgrenska Academy, University of Gothenburg, Gothenburg, Sweden; ^4^ Department of Clinical Pharmacology, Sahlgrenska University Hospital, Gothenburg, Sweden; ^5^ Department of Clinical Microbiology, Sahlgrenska University Hospital, Gothenburg, Sweden; ^6^ Department of Clinical Immunology and Transfusion Medicine, Sahlgrenska University Hospital, Gothenburg, Sweden; ^7^ Department of Laboratory Medicine, Institute of Biomedicine, Sahlgrenska Academy, University of Gothenburg, Gothenburg, Sweden

**Keywords:** SARS-CoV-2, health care workers, antibodies, neutralizing antibodies, CD4^+^ T cells

## Abstract

**Background:**

The risk of SARS-CoV-2 infection among health care workers (HCWs) is a concern, but studies that conclusively determine whether HCWs are over-represented remain limited. Furthermore, methods used to confirm past infection vary and the immunological response after mild COVID-19 is still not well defined.

**Method:**

314 HCWs were recruited from a Swedish Infectious Diseases clinic caring for COVID-19 patients. IgG antibodies were measured using two commercial assays (Abbot Architect nucleocapsid (N)-assay and YHLO iFlash-1800 N and spike (S)-assays) at five time-points, from March 2020 to January 2021, covering two pandemic waves. Seroprevalence was assessed in matched blood donors at three time-points. More extensive analyses were performed in 190 HCWs in September/October 2020, including two additional IgG-assays (DiaSorin LiaisonXL S1/S2 and Abbot Architect receptor-binding domain (RBD)-assays), neutralizing antibodies (NAbs), and CD4^+^ T-cell reactivity using an in-house developed *in vitro* whole-blood assay based on flow cytometric detection of activated cells after stimulation with Spike S1-subunit or Spike, Membrane and Nucleocapsid (SMN) overlapping peptide pools.

**Findings:**

Seroprevalence was higher among HCWs compared to sex and age-matched blood donors at all time-points. Seropositivity increased from 6.4% to 16.3% among HCWs between May 2020 and January 2021, compared to 3.6% to 11.9% among blood donors. We found significant correlations and high levels of agreement between NAbs and all four commercial IgG-assays. At 200-300 days post PCR-verified infection, there was a wide variation in sensitivity between the commercial IgG-assays, ranging from <30% in the N-assay to >90% in the RBD-assay. There was only moderate agreement between NAbs and CD4^+^ T-cell reactivity to S1 or SMN. Pre-existing CD4^+^ T-cell reactivity was present in similar proportions among HCW who subsequently became infected and those that did not.

**Conclusions:**

HCWs in COVID-19 patient care in Sweden have been infected with SARS-CoV-2 at a higher rate compared to blood donors. We demonstrate substantial variation between different IgG-assays and propose that multiple serological targets should be used to verify past infection. Our data suggest that CD4^+^ T-cell reactivity is not a suitable measure of past infection and does not reliably indicate protection from infection in naive individuals.

## Introduction

One and a half years have passed since the World Health Organization (WHO) declared Severe Acute Respiratory Syndrome 2019 Coronavirus 2 (SARS-CoV-2), which causes the clinical disease Coronavirus Disease 2019 (COVID-19), a pandemic. The risk of SARS-CoV-2 infection among health care workers (HCWs) has been a concern due to experiences from infections with two previous coronaviruses: HCWs constituted over 21% of individuals infected with Severe Acute Respiratory Syndrome (SARS) in 2002–2003 (1), and up to 29% with Middle East Respiratory Syndrome (MERS) in 2014 (2). Several studies have shown high levels of SARS-CoV-2 infection among HCWs, and a number have compared seropositivity in HCWs and the general population (3–7). However, few studies have adequate comparisons with sex and age-matched healthy controls, using the same antibody assays and during the same time periods.

Seroconversion is considered an important measure of past infection on a group level but may be unreliable for the individual. The most common target proteins in commercial antibody assays are the nucleocapsid (N), a structure within the viral particle, and the spike (S), a glycoprotein on the viral surface involved in the binding to the host cell *via* the receptor binding domain (RBD) (8). IgM antibodies, indicative of an acute virus infection, are not reliably detected in serum of patients during and/or after SARS-CoV-2 infection, and is therefore not considered a suitable measure of acute or past infection (9). While secretory-IgA is important in the mucosal immune response in SARS-CoV-2 infection (9, 10), serum-IgA is mainly derived from the bone marrow and thus not considered a surrogate measurement of secretory-IgA responses (11). The longevity of serum-IgA post infection varies between different studies: seroreversion has been observed within 3 months (9, 12), though other studies have shown that IgA may remain detectable over 6 months and up to a year post infection (13–18). Serum-IgA appears earlier than serum-IgG, but has been observed to be less long-lasting than serum-IgG post infection (9, 12, 15, 19). Serum-IgG is considered the clinical standard serological assay for detection of past infection and has been shown present up to 13 months post infection (19). However, as the sensitivity and specificity of different IgG-assays targeting the different viral structures vary, seroconversion in commercial IgG assays may be difficult to interpret in the absence of PCR testing and in asymptomatic individuals. Moreover, the potential protective role of pre-existing cross-reactive antibodies specific for the endemic coronaviruses remains to be better explored (16, 20–22).

While the commercial IgG-assays used in this study measure antibody binding to specific viral proteins, neutralizing antibody (NAb) assays measure the functional ability of the total antibody repertoire to neutralize the virus regardless of antibody class. Even though a correlate of protection for COVID-19 is not fully determined, NAbs are likely highly important for efficient protection against reinfection (23, 24). Further, some studies suggest that NAbs may be detected in all patients with mild and asymptomatic COVID-19, even in the early convalescent phase (25, 26).

It has been hypothesized that T-cell immunity may confer a more long-lasting immunity than circulating serum antibodies. In patients infected by the closely related coronavirus SARS, IgG antibodies were undetectable in approximately half of the patients within three years (27), while memory T cells reactive to the SARS N-protein were detectable up to 17 years after infection (28). Both CD4^+^ and CD8^+^ SARS-CoV-2-reactive T cells have been observed in patients post COVID-19 (26, 29–33). In line with previous studies of SARS patients, reactive T cells have been observed in twice as many subjects of unknown SARS-CoV-2 infection status as compared to IgG antibodies (29). However, CD4^+^ and CD8^+^ SARS-CoV-2-reactive T cells have also been observed in pre-pandemic samples, indicating cross-reactivity with endemic coronaviruses (29, 32). The ability of such pre-existing corona-reactive T cells to protect against SARS-CoV-2 infection remains to be determined.

The aim of this study was to investigate the prevalence of SARS-CoV-2 infection among HCWs compared to age and sex-matched blood donors, using several parallel commercial IgG-assays at multiple sampling time-points, from early in the pandemic in Sweden until vaccine introduction 10 months later. Further, we aimed to analyze the development of neutralizing antibodies and virus-reactive CD4^+^ T-cell responses in HCWs, and to examine correlations between neutralizing antibodies and different commercial IgG-assays as well as to CD4^+^ T-cell responses. We also sought to estimate the protective capacity of pre-existing CD4^+^ T-cells reactive against SARS-CoV-2.

## Materials and Methods

### Participants and Study Procedures

All HCWs (doctors, nurses, nurse assistants and administrative staff) at the Department of Infectious Diseases, Sahlgrenska University Hospital, Gothenburg, Sweden were eligible for participation and inclusion took place between March 17^th^ 2020 and January 29^th^ 2021. The number of employees at the clinic varied between 283-298 at the different time points (temporary staff not included). HCWs who changed workplace during the study period were allowed to continue in the study and new staff were offered to participate at the remaining sampling time-points. Patients with COVID-19 were cared for at the department during the whole study period. National recommendations for infection control for the care of COVID-19 patients, including personal protective equipment, were followed. The study protocol was approved by the Swedish Ethical Review Authority (Registration number 2020-01771) and subjects were included after written informed consent.

Sweden experienced two pandemic waves during the study period: the first wave was between mid-March until mid-June 2020 and the second from mid-October 2020 to February 2021, when the study period ended. Blood samples were collected at five time points (TP): 17^th^–25^th^ of March 2020 (TP1, n = 110), 11^th^–21^th^ of May (TP2, n = 110) and 8^th^–18^th^ of June 2020 (TP3, n = 80), 21^th^ of September–8^th^ of October 2020 (TP4, n = 190), and 12^th^–29^th^ of January 2021 (TP5, n = 280) (**Table 1**). HCWs were asked to fill out a questionnaire regarding COVID-19 associated symptoms and exposure to COVID-19 patients at all sampling time-points. Serum samples in sex and age-matched blood donors were analyzed at TP2 (n = 111), TP4 (n = 181), and TP5 (n = 253).

**Table 1:** Characteristics of participants at every sampling time-point and results from the serological assays and PCR-testing.

	HCWs	HCWs	HCWs	HCWs	HCWs	BD	BD	BD
	Mar TP1	May TP2	June TP3	Oct TP4	Jan TP5	May TP2	Sep TP4	Jan TP5
	(n = 110)	(n = 110)	(n = 80)	(n = 190)	(n = 280)	(n = 111)	(n = 181)	(n = 253)
No. (%) of subjects								
Sex								
Female	82/110 (74.5)	82/110 (74.5)	68/80 (85.0)	147/190 (78.4)	226/280 (80.7)	83/111 (74.8)	142/181 (78.5)	204/253 (80.6)
Male	28/110 (25.5)	28/110 (25.5)	12/80 (15.0)	43/190 (22.6)	54/280 (19.3)	28/111 (25.2)	39/181 (21.5)	49/253 (19.4)
Age (years)								
Mean (SD)	40.7 (12.9)	40.7 (12.9)	44.7 (13.9)	42.6 (13.2)	50.5 (13.3)	39.7 (12)	42.8 (13.4)	43.5 (13.3)
Range	20–69	20–69	22–76	20–76	22–79	20–69	21–74	23–75
Positive IgG antibodies								
N+S-assay	0/110 (0.0)	7/110 (6.4)	9/80 (11.3)	31/190 (16.3)	82/280 (29.3)	4/111 (3.6)	9/181 (5.0)	30/253 (11.9)
N-assay	1/110 (0.09)	8/110 (7.2)	9/80 (11.3)	25/190 (13.2)	63/280 (22.5)	3/111 (2.7)	6/181 (3.3)	25/253 (9.9)
S1/S2-assay				35/190 (18.4)			13/181 (7.2)	
RBD-assay				39/190 (20.5)	105/280 (37.5)			48/253 (19.0)
PCR positivity	0/110 (0.0)	10/110 (9.1)	8/80 (10.0)	31/190 (16.3)	85/280 (30.4)			

BD, Blood donors; HCWs, Health care workers; PCR, polymerase chain reaction test.

No routine PCR-tests were performed in asymptomatic subjects, but all HCWs were obligated to undergo PCR-testing for SARS-CoV-2 if they had any symptoms commonly associated with COVID-19, such as dry cough, body temperature > 37.5°C, ageusia, or general malaise. In individuals with symptoms, a negative PCR-test taken 12–72 hours after symptom onset ruled out an ongoing SARS-CoV-2 infection. Verified SARS-CoV-2 infection was defined as positive PCR and/or NAbs, and/or IgG positivity in ≥ 3 commercial IgG-assays. Asymptomatic SARS-CoV-2 infection was defined as detectable seroconversion in the absence of reported symptoms with a positive PCR test.

### Real-Time Polymerase Chain Reaction (RT-PCR) Assay

Nucleic acid was extracted from pooled nasopharyngeal and throat swabs in a MagNA Pure 96 instrument using the Total Nucleic Acid isolation kit (Roche). RT-PCR targeting the RdRP region was performed in a QuantStudio 6 instrument (Applied Biosystems, Foster City, CA) as previously described (34). Cycle threshold (Ct) values < 38 were regarded as positive.

### SARS-CoV-2 Analyses at the Different Time-Points

SARS-CoV-2-specific antibodies were analyzed in serum samples using several commercially available antibody binding assays (based on N, S or RBD antigens), and an in-house virus neutralization assay at the different time-points: TP1-TP3 (N-assay and N+S-assay), TP4 (N-assay, N+S-assay, S1/S2-assay, RBD-assay and NAbs) and TP5 (N-assay, N+S-assay and RBD-assay). Serum samples from the blood donors were analyzed at TP2 (N-assay and N+S-assay), TP4 (N-assay, N+S-assay and S1/S2-assay) and at TP5 (N-assay, N+S-assay, and RBD-assay). SARS-CoV-2 specific CD4^+^ T cells were analyzed in all HCWs at TP4.

#### Commercial SARS-CoV-2 Antibody Assays

N-assay: Architect N is a semi-quantitative chemiluminescent microparticle immunoassay (Abbott Laboratories, USA), measuring IgG binding to SARS-CoV-2 N-protein. IgG index (S/CO) ≥ 1.4 was defined as positive.

N+S-assay: iFlash 1800 is a quantitative chemiluminescent immunoassay (YHLO, China), measuring IgG binding to both SARS-CoV-2 S and N-proteins. IgG concentrations ≥ 10 AU/ml were defined as positive.

S1/S2-assay: LiaisonXL (DiaSorin, Italy) is a quantitative chemiluminescence immunoassay measuring IgG binding to the S1/S2 subunits. IgG concentrations ≥ 15 AU/ml were defined as positive.

RBD-assay: Architect S (Abbott Laboratories, USA) is a quantitative chemiluminescent microparticle immunoassay measuring IgG binding to the RBD of the S-protein. IgG concentrations ≥ 7.1 binding antibody unit (BAU)/ml were defined as positive.

#### SARS-CoV-2 Neutralizing Antibody Assay

After inactivation of complement in serum for 30 minutes at 56°C, NAbs were determined by incubating 25μL of 2-fold dilutions (1/2–1/256) of each serum in fetal-calf serum free DMEM with 25 μL of 100TCID_50_ of SARS-CoV-2 (DE strain, isolated from sample collected February 25, 2020) in duplicate for two hours at 37°C. Thereafter the serum/virus mixture was added to confluent Vero cells (ATCC CCL-81) in 96-well microtiter plates with 175 μL DMEM with 2% inactivated fetal calf serum and incubated at 37°C in at 5%CO_2_. Ten-fold serial dilutions of the virus, 10–1,000 TCID_50_, were added in duplicate to separate wells as an infection control. The plates were examined after 72 hours, using an inverted microscope, and complete cytopathic effect (CPE) was determined. The presence of any CPE of the cells was then recorded in the wells and the titer of the sera was calculated as previously described (35). Sera with antibody titers >4 were considered neutralizing, confirming the presence of antibodies with capacity to block infection. Sera (n = 17) from patients and blood donors from before December 2019 were used as negative controls and no neutralization was detected in any of these samples.

#### SARS-CoV-2-Specific CD4^+^ T-Cell Responses

T-cell analysis was performed using an in-house *in vitro* assay based on peptide stimulation of cells in whole blood and evaluation of the expression of the activation markers CD25 and OX40 (CD134) on the cell surface of CD4^+^ T cell or CD69 and CD137 on CD8^+^ T cells by flow cytometry, modified from a previously described protocol (36, 37). Briefly, 250µl whole blood was diluted 1:1 with RPMI alone or containing a peptide pool based on the S1-domain of the SARS-CoV-2 S protein (130-127-041) or a mixture of SMN peptide pools covering the S1 domain, S C-terminal (130-126-700), Membrane (130-126-702) and N (130-126-698) proteins (all from Miltenyi Biotech, Bergisch Gladbach, Germany). The final concentration of peptides was 0.3 nmol/ml per peptide pool. All samples were also stimulated with phytohemagglutinin (PHA) as a positive control (final concentration 10 µg/ml). After mixing, cells were incubated for 48 h at 37°C in 5% CO_2_. After the incubation, 250 µl of the supernatants were removed, samples were fixed through the addition of 50 µl TransFix (Cytomark, Buckingham, UK) and cells kept at +4°C until flow cytometric staining was performed by adding anti-CD3-V450 (#560365), anti-CD4-BV605 (#562658), anti-CD8-BV510 (#563919), anti-CD25-PE (#555432), anti-CD134-PECy7 (#563663), anti-CD69-FITC (#555530) and anti-CD137-APC (#550890) antibodies to the pre-fixed cells (all antibodies from BD Biosciences, Franklin Lake, NJ). Red blood cells were lysed using ammonium chloride in an automated BD FACS Lyse Wash Assistant program and analyzed on a FACSLyric flow cytometer (both from BD Biosciences). FMO controls were run regularly using PHA-stimulated cells to ensure that correct gates were used to detect cells co-expressing CD25 and OX40 or CD69 and CD137. Values for the percentage of cells reactive against S1 and SMN peptide pools was calculated by subtracting values for non-stimulated cells from values when cells were incubated with the respective peptide pool.

### Statistical Analysis

Differences between groups were analyzed with Fisher**’**s exact test. Agreements were analyzed using kappa analysis and associations using Spearman**’**s correlation analysis. ROC analyzes were used to evaluate the performance of the assays. P < 0.05 was considered statistically significant. Statistical analyses were performed using GraphPad Prism 8 (GraphPad Software, Inc).

## Results

### Participants

A total of 314 HCWs were recruited. Of these, 113 participated at one sampling time-point, 26 at two, 79 at three and 96 at four time-points (**Supplementary Figure 1**). TP3 was arranged as an extra time-point for additional HCWs who had not entered the study at TP1 or TP2, thus no HCW participated at all five time-points. At TP4, 190 HCWs participated, and the most extensive analyses were performed, including NAbs and T-cell reactivity. At the last sampling time-point (TP5), 280 HCWs participated, including 166 of the 190 HCWs at TP4. A total of 778 PCR tests (mean 2.5 per subject, range 0–10) were analyzed during the entire study period. Serum samples collected from PCR-positive subjects outside of the sampling time-points were also included, resulting in a total of 882 samples (mean 2.8 per subject, range 1–8) analyzed during the study period. The majority of the HCWs were female (255/314, 81.2%), with an age span of 20–79 years (mean 42.6).

### SARS-CoV-2-Specific IgG Antibodies in HCWs and Blood Donors

**Figure 1:**
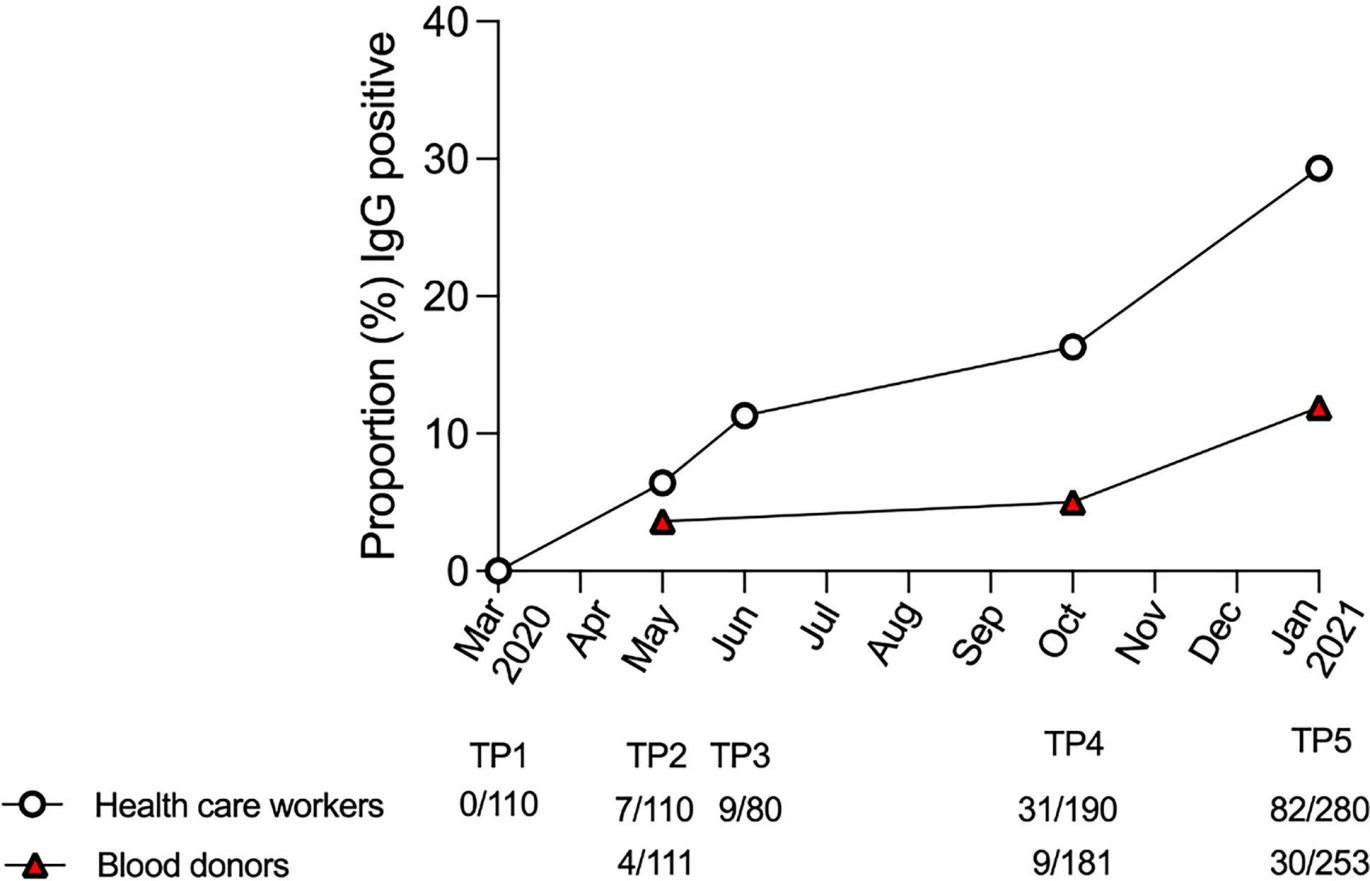
IgG positivity in the N+S assay in health care workers and blood donors over time. Proportion of IgG-positive health care workers (white circles) and blood donors (red triangles), as measured with the N+S-assay, at each sampling time-point (TP) from March 2020 to January 2021.

The proportion of IgG-positive HCWs increased from 0.0% in March 2020 (TP1), to 6.4% in May (TP2), 11.3% in June (TP3), 16.3% in September/October (TP4), and 29.3% in January 2021 (TP5), as measured with the N+S-assay (**Table 1** and **Figure 1**). For the matched blood donors, the proportion of IgG-positive individuals was consistently lower compared to HCWs at all three time-points: 3.6% at TP2, 5.0% at TP4, and 11.9% at TP5 (**Table 1** and **Figure 1**). Similar results were found using the S1/S2-assay at TP4 and the RBD-assay at TP5. The differences in proportions of IgG-positive HCWs and blood donors was statistically significant at both TP4 (p < 0.001) and TP5 (p < 0.0001).

### HCWs With Verified COVID-19

Verified COVID-19 was defined as a positive PCR-test and/or NAbs, or IgG positivity in ≥ 3 commercial IgG-assays. The percentage of HCWs that had a positive PCR-test increased from 0.0% at TP1 to 16.3% at TP4. Positive PCR-tests were performed on average 142 days (range 88–203) prior to the analysis at TP4. By TP5, 10 months from study start and in conjunction with the start of COVID-19 vaccination, 30.4% (85/280) had tested PCR-positive (**Table 1**).

NAbs were analyzed in HCWs at TP4, at which point 18.4% (35/190) had a neutralizing titer. Four study subjects with verified COVID-19 at TP4 had not developed NAbs. One asymptomatic subject lacking both a positive PCR and NAbs was positive in three out of four commercial IgG-assays at TP4, hence defined as verified infection. This resulted in 20.5% (39/190) of study subjects defined as having had verified COVID-19 at TP4. Of these 39 subjects, 21 were positive in all 5 antibody assays including NAbs, while 13 were positive in all antibody assays, PCR and CD4^+^ T cell reactivity. There were no substantial differences in sex and age in HCWs with or without verified infection (**Table 2**).

**Table 2:** Characteristics of health care workers with and without verified SARS-CoV-2 infection at time-point 4 (21^th^ of September–8^th^ of October 2020) and specificity and sensitivity of all SARS-CoV-2 specific assays in time-point 4 (commercial IgG-assays, neutralizing antibodies and CD4^+^ specific T cells).

	All health care workers	Verified SARS-CoV-2^a^	No SARS-CoV-2^a^
	(n = 190)	(n = 39)	(n = 151)
Characteristics No. (%) of subjects			
Sex			
Female	147/190 (77.4)	34/39 (87.2)	113/151 (74.8)
Male	43/190 (22.6)	5/39 (12.8)	38/152 (25.2)
Age (years)			
Mean (SD)	42.9 (13.3)	38.9 (12.6)	43.5 (13.2)
Range	20–76	24–66	20–76
Occupation			
Physician	45/190 (23.7)	3/39 (7.7)	42/152 (27.8)
Nurse	93/190 (48.9)	22/39 (56.4)	71/152 (47.0)
Assistant Nurse	32/190 (16.8)	13/39 (33.3)	19/151 (12.6)
Administrative staff	16/190 (8.4)	1/39 (2.6)	15/151 (9.9)
Other^b^	4/190 (2.1)	0/39 (0)	4/151 (2.6)
SARS-CoV-2 PCR			
Positive	31/190 (16.3)	31/38 (81.6)	0/105 (0.0)
Negative	112/190 (58.9)	7/38 (18.4)	105/105 (69.5)
Not performed	47/190 (24.7)	1/39 (2.6)	46/151 (30.5)
SARS-CoV-2 Serology			
Neutralizing antibodies			
Positive	35/190 (18.4)	35/39 (89.7)	0/151 (0.0)
Negative	155/190 (81.5)	4/39 (10.3)	151/151 (100)
N-assay			
Positive	25/190 (13.2)	24/39 (61.5)	1/151 (0.7)
Negative	165/190 (86.8)	15/39 (38.5)	150/151 (99.3)
S1/S2-assay			
Positive	34/190 (17.9)	29/39 (74.4)	5/151 (3.3)
Negative	156/190 (82.1)	10/39 (25.6)	146/151 (96.6)
RBD-assay			
Positive	39/190 (20.5)	36/39 (92.3)	3/151 (2.0)
Negative	151/90 (79.5)	3/39 (7.7)	148/151 (98.0)
N+S-assay			
Positive	31/190 (16.3)	31/39 (79.5)	0/151 (0.0)
Negative	159/190 (83.7)	8/39 (20.5)	151/151 (100)

a Verified infection with either positive SARS-CoV-2 PCR (CT value < 38), positive neutralizing titer (> 4) and/or IgG-positivity in ≥ 3 commercial IgG-assays.

b Laboratory personnel, social worker, it-manager.

Of the 31 study subjects with a positive PCR-test at TP4, all had experienced mild symptoms. The remaining eight individuals (20.5%) with verified COVID-19 lacked a positive PCR-test and were considered to have had an asymptomatic infection. Of the asymptomatic subjects, 87.5% (7/8) were positive in NAbs compared to 90% (28/31) of those with PCR-positive verified infection.

### SARS-CoV-2-Specific IgG Antibodies in HCWs With and Without Verified Infection

**Figure 2:**
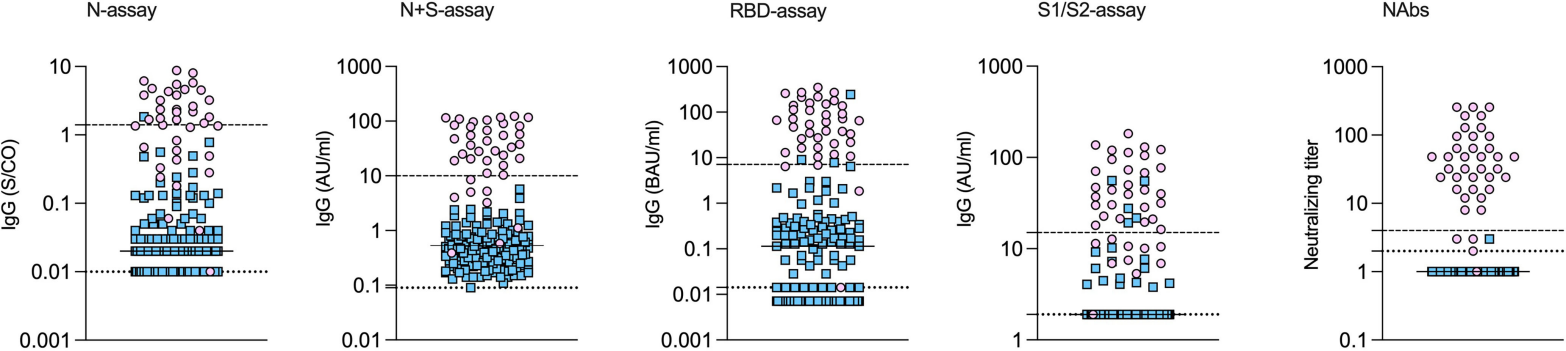
SARS-CoV-2 specific antibodies measured by different assays in health care workers with and without verified COVID-19. Health care workers with (pink circles) and without (blue squares) verified COVID-19. Concentrations of IgG measured by different commercial IgG-assays and by virus neutralization at time-point 4. Medians indicated by horizontal lines. The upper dashed lines indicate the cut-off for positivity, and the lower dotted lines indicate the lowest detectable concentration/index.

**Figure 3:**
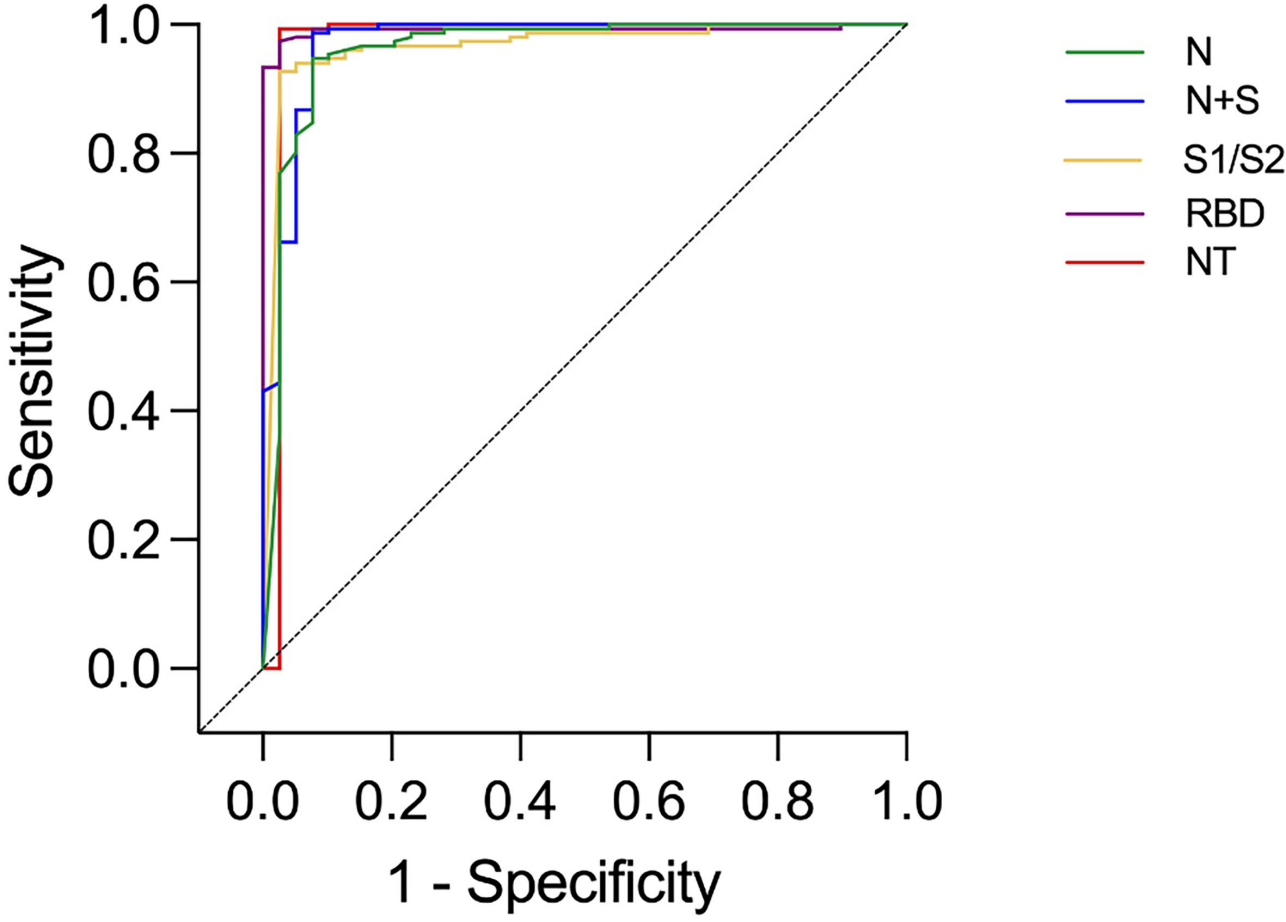
ROC curves presenting results from different commercial IgG-assays and neutralizing antibody titers (NT) in health care workers with and without verified infection at time-point 4. Identity line (diagonal).

**Figure 4:**
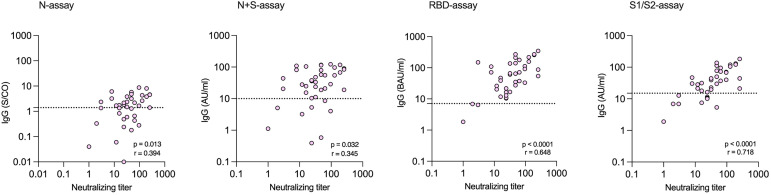
Correlations between levels of neutralizing antibodies (NAbs) and levels of SARS-CoV-2 specific IgG measured at time-point 4 by commercial assays among health care workers with verified infection. Dotted lines indicate the cut-off for positivity in each commercial IgG-assay.

Among the 39 study subjects defined with verified COVID-19 by TP4, 61.5% were positive in the N-assay and 79.5% in the N+S-assay, based on suggested clinical thresholds. Additionally, 74.4% were positive in the S1/S2-assay and 92.3% in the RBD-assay at TP4 (**Table 2** and **Figure 2**). ROC analyses of the five antibody assays resulted in area under the curve (AUC) ranging from 0.96-0.99 (**Figure 3**). Thirty-five of the subjects with verified COVID-19 at TP4 had NAbs. Of these, 23 (65.7%) were positive in the N-assay, 29 (82.9%) in the N+S-assay, 29 (82.9%) in the S1/S2-assay, and 35 (100%) in the RBD-assay. The agreement between NAbs and the four commercial IgG-assays ranged from almost perfect to substantial, with kappa indexes ranging from 0.93 in the RBD-assay, 0.85 in the N+S-assay, 0.81 in the S1/S2-assay and 0.75 in the N-assay. Furthermore, in HCWs with verified COVID-19, correlations between NAbs and IgG levels as measured in all four commercial IgG-assays were statistically significant (**Figure 4**).

Of the four NAb-negative subjects with verified infection, three were PCR-positive but lacked detectable IgG in three to four of the four commercial IgG-assays (Patients 1-2, 4 **Table 3**), while the fourth asymptomatic subject was positive in three of the commercial IgG-assays (Patient 3, **Table 3**). For the three PCR-positive subjects without NAbs, mean 154 days (range 107-180) had passed after the PCR-verified infection, compared to mean 140 days (range 88–203) in subjects with NAbs. Previous blood samples from all three subjects were analyzed for prior detectable NAbs, at days 27, 71 and 71 days after PCR-positivity, respectively. NAbs were detectable in one of these three subjects (Patient 4, **Table 3**) in a blood sample 27 days after PCR-positivity.

**Table 3:** Health care workers defined with verified* COVID-19 yet without detectable neutralizing antibodies (NAbs) and/or IgG in the N and N+S-assay at time-point 4 (21^th^ of September–8^th^ of October 2020).

	Patient 1	Patient 2	Patient 3	Patient 4	Patient 5
Sex	Female	Female	Female	Female	Female
Age (years)	25	28	58	62	44
Days post pos PCR	175	180	PCR neg	107	203
Former IgG response in N+S-assay	+	–	+	+	–
NAbs^a^	–	–	–	(-)	+
					
SARS-CoV-2 IgG					
N-assay	–	–	+	–	–
N+S-assay	–	–	+	+	–
S1/S2-assay	–	–	–	–	–
RBD-assay	–	–	+	–	+
CD4^+^ S1	+	+	+	+	+
CD4^+^ SMN	+	–	+	+	+

* = defined as positive PCR and/or NAbs or positivity in ≥ 3 commercial IgG-assay.

a (-) = positivity found in blood sample from earlier convalescence phase.

Two subjects with verified infection lacked IgG in both the N and N+S-assay, despite repeated serum samples being analyzed (**Supplementary Figure 2**). Both had multiple positive PCR tests with lowest CT values of 16 and 20, respectively. One had NAbs and was IgG-positive in the RBD-assay, while the second was negative in all serological assays (Patients 2 and 5, **Table 3**). Of the remaining 29 PCR-positive subjects by TP4 and/or TP5, an additional five had lost positivity in the N and N+S assay at this time point. There were no apparent differences in antibody kinetics among these subjects when studied over time post PCR-positivity (**Supplementary Figure 2**).

Among the HCWs with no verified infection, none were positive in the N+S-assay, 0.7% were positive in the N-assay, 2.0% in the RBD-assay, and 3.3% in the S1/S2-assay (**Table 2** and **Figure 2**).

Of the 151 HCWs with no verified infection at TP4, 131 also participated at TP5. Of these, 27 tested PCR-positive between TP4 and TP5, of which one had tested positive in the RBD-assay alone at TP4. Additionally, one HCW with no verified infection but who was positive in both the RBD-assay and the S1/S2-assays at TP4, developed a 13-times higher IgG concentration in the RBD-assay and became strongly positive in the N and the N+S-assay at TP5, thus likely had asymptomatic SARS-CoV-2 infection after TP4.

### Antibody Persistence in Commercial IgG-Assays

The duration of IgG-positivity was determined with the N and N+S-assays as these were used at all five time-points. Of the 39 subjects with verified infection at TP4 (88–203 days post PCR positivity in the 31 symptomatic subjects), 37/39 (94.9%) had at some time-point detectable antibodies in the N and N+S-assays. Thirty-five of these 39 subjects also participated at TP5, approximately 100 days later. N-specific IgG decreased during this time: 62.9% (22/35) were positive in the N-assay at TP4 and 28.6% (10/35) at TP5. In the combined N+S-assay, the proportion with a positive test fell from 80.0% (28/35) at TP4 to 62.9% (22/35) at TP5. S-specific IgG was more durable and the proportion HCWs positive in the RBD-assay was stable: 91.4% (32/35) at TP4 and 94.3% (33/35) at TP5, as one subject who was negative in the RBD-assay 175 days post PCR positivity was weakly positive 291 days post PCR positivity.

### SARS-CoV-2-Reactive CD4^+^ T-Cell Responses in HCWs With and Without Verified COVID-19

**Figure 5:**
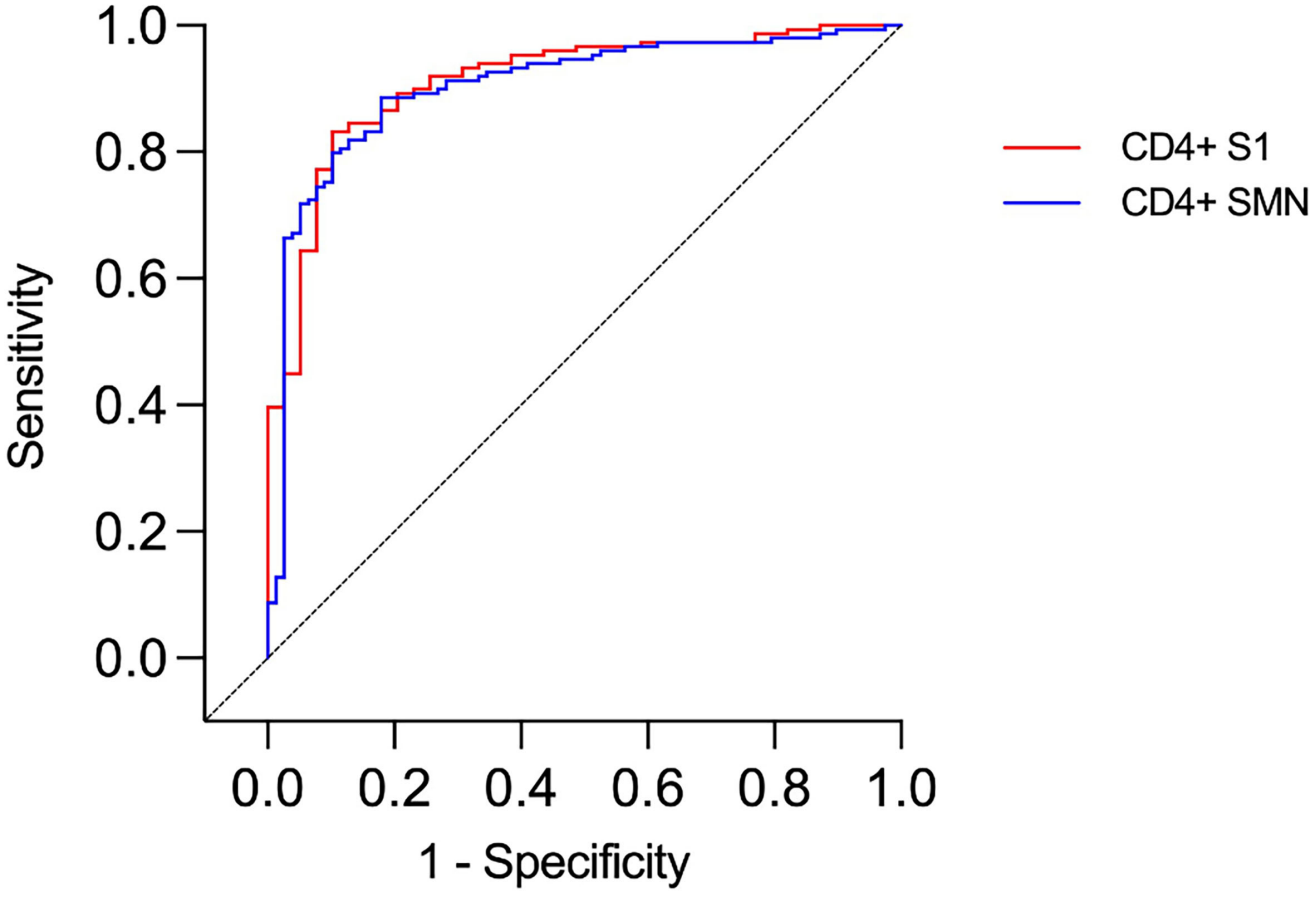
ROC curves presenting results from CD4^+^ T cell assays in health care workers with and without verified infection. Identity line (diagonal).

**Figure 6:**
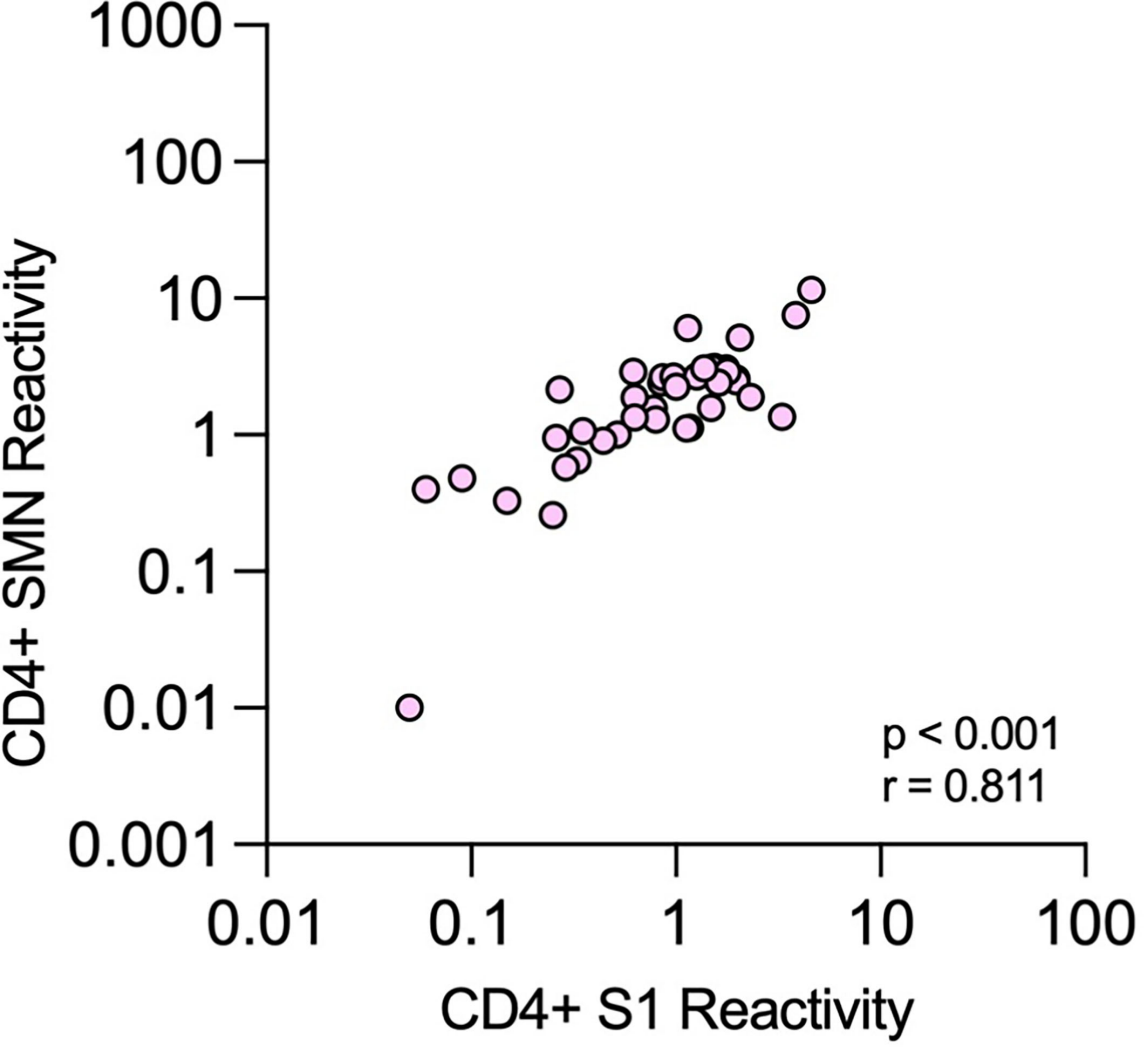
Correlation between CD4^+^ T-cell reactivity against S1 and SMN peptide pools. Correlations between proportions of OX40^+^CD25^+^ cells among all CD4^+^ T cells after stimulation with the two different peptide pools at time-point 4 in health care workers with verified COVID-19.

The presence of T cells reactive against SARS-CoV-2 was tested using an assay modified from Zaunders *et al.* that measures the proportion of CD4^+^ and CD8^+^ T cells that express the activation markers CD25 and OX40 or CD69 and CD137, respectively, after *in vitro* stimulation (36, 37). The assay was used to analyze samples from 188 of the 190 HCWs at TP4. When ROC curves were constructed for CD4^+^ T cell data, the AUC was over 0.9 for both peptide pools (0.91 and 0.90, respectively, **Figure 5**). As expected, there were substantial agreements between S1 and SMN reactivity (kappa 0.7). Furthermore, there was a significant correlation between S1 and SMN CD4^+^-reactivity among those HCWs with verified infection at TP4 (p < 0.0001, r = 0.81; **Figure 6**). When CD8^+^ T cell data were considered, the AUC of the ROC curves were 0.582 for S1 and 0.657 for SMN; further CD8^+^ T cell data analysis was therefore not performed.

Cutoff values giving both 95% specificity and sensitivity for the CD4^+^ T-cell analysis could not be attained. The maximal value for Youden’s J statistics was reached with cutoffs of ≥ 0.25% positive CD4^+^ cells for S1 (Youden index 72,97) and ≥ 0.9% for SMN (Youden index 70,64). For these cutoffs, a sensitivity of 89.7% and specificity of 83.2% was obtained for S1, and a sensitivity of 82.1% and specificity of 88.6% was obtained for SMN. Using these cutoffs, 35 (S1) or 32 (SMN) of the 39 subjects with confirmed infection were positive, while 25 (S1) or 17 (SMN) of 149 subjects without confirmed infection were positive. In total, 67/188 (36%) had positive reactivity in at least one of the two assays. Of those with verified infection, 90% (35/39) had positive S1 or SMN reactivity compared to only 21% (32/149) of those without verified infection.

**Figure 7:**
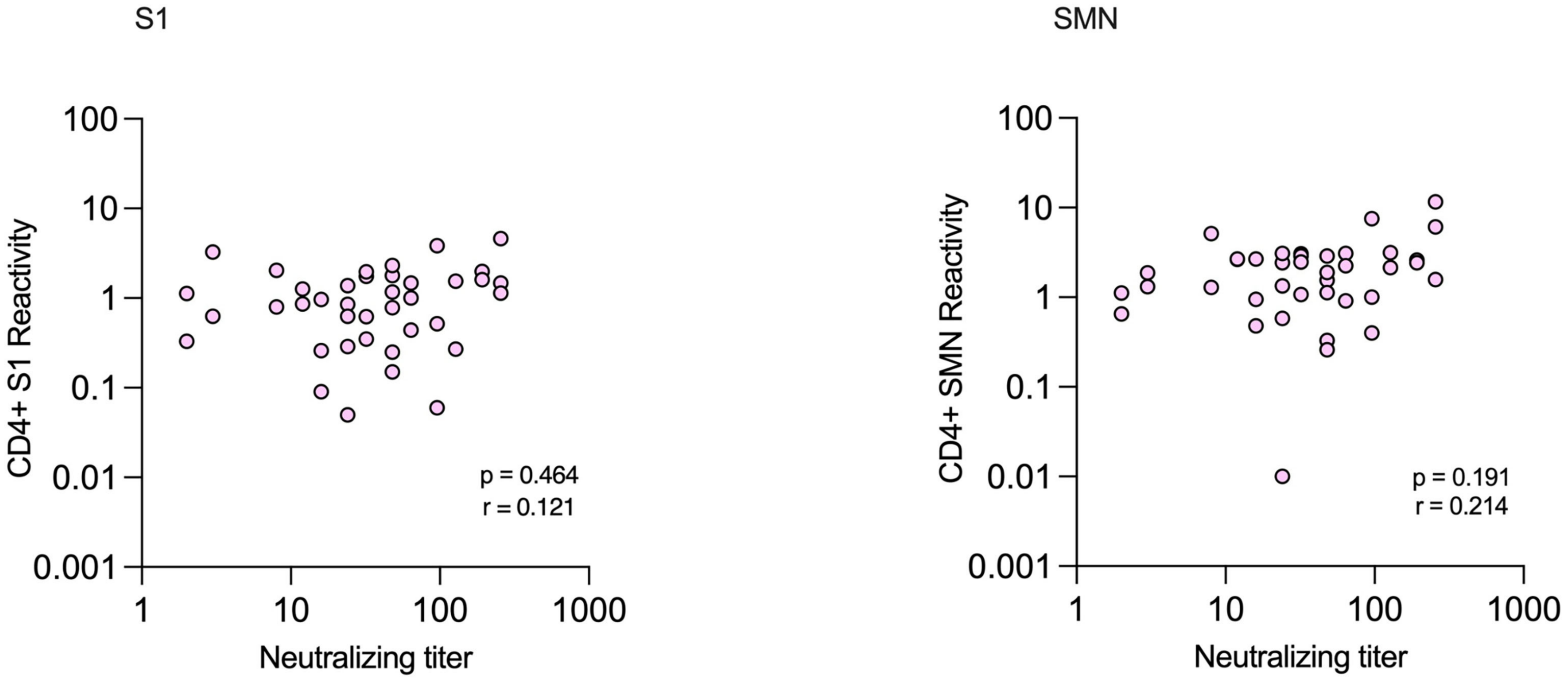
Correlations between CD4^+^ T-cell reactivity against S1 and SMN peptide pools and neutralizing antibody titers in health care workers with verified COVID-19.

Of the subjects with NAbs, 31/35 (89%) had positive reactivity against S1 or SMN while 29/35 (83%) had positive reactivity against both peptide pools. There were no significant correlations between levels of NAbs and CD4^+^-reactivity against either S1 or SMN (**Figure 7**). All four subjects with verified infection who lacked NAbs had positive CD4^+^-reactivity against at least one of the peptide pools (Patients 1-4, **Table 3**).

### Analysis of Infection Rate During Second Pandemic Wave

The extensive immune analyses performed at TP4 preceded a second wave of infections, enabling estimations of the protective ability of pre-existing immune responses. None of the 35 subjects with verified infection at TP4 who participated at TP5 suffered a reinfection. Of the 129 HCWs with no verified infection at TP4 who participated at TP5, 27 demonstrated CD4^+^-reactivity against either S1 or SMN at TP4, based on the thresholds calculated based on Youden’s J statistics. Of these, 26% (7/27) subsequently tested PCR-positive for SARS-CoV-2 between TP4 and TP5: 6/27 showed reactivity against S1, 3/27 against SMN and 2/27 against both S1 and SMN. In comparison, 20% (20/102) of HCWs lacking detectable CD4^+^-reactivity against either S1 or SMN at TP4 became infected, resulting in an odds ratio of 1.4 (95% confidence interval 0.6-3.6, p = 0.59) for HCWs with CD4^+^-reactivity compared to HCWs lacking CD4^+^-reactivity becoming infected.

## Discussion

This longitudinal study spans from the very beginning of the COVID-19 pandemic, less than three weeks after the first confirmed case in Gothenburg, until the start of vaccinations of HCWs 10 months later. During this time, seroprevalence among HCWs increased from 0% in March 2020 to over 30% in January 2021. The proportion of seropositive HCWs was almost twice that of age and sex-matched blood donors in May 2020, and close to triple the number in January 2021. Seroprevalence estimates in HCWs have varied widely in different studies (38), which likely reflect the geographical and temporal differences in disease burden during data collection. Studies contemporaneously comparing seroprevalence in HCWs and the general population have largely shown that HCWs have suffered a higher incidence of COVID-19 (4, 6, 39, 40).

There are many reasons why HCWs run a higher risk of SARS-CoV-2 infection. Although Sweden has not implemented a strict lockdown, all citizens have been encouraged to work from home when possible. As this is not feasible for HCWs, they have an increased risk of exposure which is not only related to patient care. Another Swedish study, investigating seroprevalence among university employees, found that HCWs working in COVID-19-units had three times higher seroprevalence compared to non-health care employees (18% vs 6%) (5). Surprisingly, no significant difference in seroprevalence was observed between HCWs in non-COVID-19-units and non-health care university employees, demonstrating that the risk of infection is indeed higher when working directly with COVID-19 patients, which is in line with other studies (7, 39). In the present study, approximately 90% of the HCWs had contact with COVID-19 patients during the study period (**Table 2**). Additional parameters have been more highly associated with SARS-CoV-2 infection among HCWs than caring for COVID-19 patients in some studies, such as age, exposure *via* house-hold contacts and household size (5, 38, 41–43).

It has been suggested that as many as 40–45% of all COVID-19-infections are asymptomatic (44, 45). In our study, which carefully followed a group of hospital employees of relatively low age, only 21% of those with verified infection were defined as asymptomatic, suggesting that a total lack of symptoms is lower than these previous estimates. As all HCWs were required to be PCR-tested when they had any symptoms suggestive of COVID-19, we were able to serologically identify asymptomatic cases, reducing the risk of recall bias which is a possible confounder when using self-reported data. Furthermore, since multiple assays were used to analyze antibodies in all HCWs, we were able to identify asymptomatic subjects with high accuracy. Of the eight asymptomatic subjects with verified infection, only one lacked NAbs. Thus, NAbs were positive in most (88%) asymptomatic study subjects, equivalent to the proportion of NAb-positive subjects with PCR-confirmed infection (90%). This is in line with previous studies of HCWs with mild and asymptomatic SARS-CoV-2 infection, with around 90% testing positive in NAbs > 100 days post IgG or PCR-confirmed infection (14, 30, 46).

Seow et al. showed neutralizing titers approaching baseline within 80 days post symptom onset in 4/31 HCWs with mild or asymptomatic COVID-19 (47). When analyzing NAbs in blood samples from the earlier convalescent phase in the 3/31 PCR-positive subjects lacking NAbs at TP4 in our study, one was indeed found to have been NAb-positive 27 days after positive PCR-test. Therefore, it is possible that the two other subjects also had NAbs that could have been detected if blood samples had been collected and analyzed earlier after infection from these individuals. Moreover, these three subjects were also negative in three to four of the four commercial IgG-assays. However, evidence of T cell-reactivity was apparent in all three subjects, as well as in the third and asymptomatic subject who lacked NAbs. At the last sampling time-point, approximately 100 days later and after start of the second wave, we could confirm that none of these three had been re-infected, nor any of the other subjects with previous verified infection.

Anti-S IgG-assays have been proposed to be superior to anti-N IgG-assays for detecting mild COVID-19 in the late convalescent phase. For example, Havervall et al. found that only 68% of previous IgG-positive study subjects were positive in an N-assay after four months follow-up, while 98% were still positive in an S-assay (46). We followed the IgG response in separate IgG-assays in HCWs post asymptomatic and mild COVID-19 up to 300 days after positive PCR test. Positivity in the N-assay was indeed markedly lower in the later phases, with less than two thirds of HCWs being positive 88–203 days post PCR-positivity at TP4, and less than one third around 206–323 days post PCR-positivity among these same study subjects at TP5. In comparison, positivity in the combined N+S-assay was 80% at TP4 and almost 63% at TP5, and over 90% tested positive in the RBD-assay at both these time-points.

RBD-specific antibodies have been described as having the best correlation with NAbs (48), which is in line with our findings that the RBD-assay, as well as the S1/S2-assay, correlated best to NAbs. However, while 100% of subjects with NAbs were also positive in the RBD-assay, less than 90% of the RBD-positive study subjects were positive in NAbs. Importantly, 2/4 subjects who were weakly RBD-positive, yet NAb-negative at TP4, most likely became infected after TP4 (one became PCR-positive and the other seroconverted in all assays analyzed at TP5). Many studies have suggested that neutralization capacity can be accurately measured by RBD-IgG binding assays, but our results demonstrate the risks of relying on proxy measures of antibody neutralization.

T-cell reactivity can also be determined by different methods, making comparisons between studies relatively difficult. In this study, we stimulated whole blood samples with overlapping peptides from the S1 part of the S protein or a mixture of overlapping peptides from the S1, S, C-terminal, M and N proteins and analyzed the *in vitro* induced expression of the activation markers CD25 and OX40 on CD4^+^ helper T cells as a reflection of antigen-specific cell activation. This method captures a majority of antigen-specific CD4^+^ T cells, independently of their expression of cytokines or other effector molecules, thereby enabling highly sensitive detection of antigen-specific T cells with potentially heterogenous effector functions (36). However, these markers are only suitable for detection of activated CD4^+^ T cells but not CD8^+^ reactivity. We attempted to measure CD8^+^ reactivity by instead using CD69 and CD137 as activation markers, which did not give sufficiently good separation between infected and non-infected individuals. This may not only reflect a lower sensitivity of the assay, but also that CD8^+^ responses appear to specifically target epitopes outside of the peptide pools used here, most notably within non-structural proteins derived from the ORF1ab part of the virus (49–52).

While CD8^+^ T cells recognize and kill host cells already infected by virus, CD4^+^ T cells influence other cells and are required for the germinal center reaction that is necessary for production of high-quality, protective antibodies and memory B cells. For the cut-offs defined here, more than 90% of subjects with verified infection had CD4^+^ reactivity to SARS-CoV-2 peptide pools S1 and/or SMN at TP4. However, this was determined in a group of rather young individuals, all of which had mild or even asymptomatic disease. It is possible that individuals that have had more severe disease will develop higher reactivity, but also that the young age of the study participants influenced the strength of the immune response. Nevertheless, the proportion of subjects with verified infection that developed CD4^+^ T-cell responses is in line with previous studies showing that most COVID-19 patients develop such responses (26, 29, 30). In the present study, the agreements between NAbs and CD4^+^ T-cell reactivity to S1 and SMN were modest, and we saw no significant correlations between the levels of NAbs and frequencies of T cells reacting with S1 or SMN. Previous studies have also shown discordant results between NAbs and CD4^+^ T-cell responses depending on peptid pool used (30), while in this study, only 11% of those with NAbs did not have CD4^+^ T-cell reactivity to either S1 or to SMN.

Over a third of all HCWs showed any T-cell reactivity at TP4, including 21% (32/149) of those without any verified infection. Importantly, 26% of subjects with pre-existing CD4^+^ T-cell reactivity went on to develop PCR-verified infection by TP5, compared to 20% of subjects without pre-existing CD4^+^ T-cell reactivity. Several other studies have found pre-existing CD4^+^ T-cell reactivity against peptides from SARS-CoV-2 in the absence of previous infection, and it has been hypothesized that these may confer some protection (51, 53–55).

There is a paucity of studies measuring pre-existing CD4^+^ T cell immunity in a cohort of non-infected individuals and then following their infection rate over time (56). Although the sample size is small, our data suggest that detectable CD4^+^ T-cell reactivity is a less suitable measure of past infection than S-specific antibody levels and further, does not reliably indicate protection from infection.

This study has several limitations. Not all HCWs participated at all sampling time-points and the assays differed between the time-points, with NAbs and T-cell reactivity analyzed at only one time-point. Mucosal samples were not available, making it possible that asymptomatic subjects who may have only developed secretory-IgA were misclassified as uninfected. IgM and IgA seroconversion was not analyzed, thus the full serological response to infection was not presented. CD8^+^-reactivity was not detected using the selected assay, thus the full T-cell mediated immune responses were not measured. The age and sex-distribution of our cohort, while representative of HCWs, is not representative of the general population. Similarly, the blood donor comparison group may not ideally represent the general population.

### Conclusions

HCWs working with COVID-19 patients in Sweden have been infected with SARS-CoV-2 to a larger extent compared to matched blood donors, from the beginning of the pandemic until the introduction of COVID-19 vaccinations. We demonstrate the difficulty in interpreting results from single IgG-assays and hence the benefits of using multiple serological targets to be able to verify past infection. We show that NAbs are still detectable in most patients more than three months after mild COVID-19, with high levels of NAbs still detected after 200 days post infection which correlate strongly with results from commercial IgG-assays targeting both the N and the S-proteins. Lastly, pre-existing CD4^+^ T-cell reactivity was present in similar proportions in subjects who subsequently became infected in the pandemic’s second wave as those that did not, suggesting that the presence of such cells in the absence of NAbs is not a reliable indicator of protection in previously non-infected individuals.

## Data Availability Statement

The raw data supporting the conclusions of this article will be made available by the authors, without undue reservation.

## Ethics Statement

The studies involving human participants were reviewed and approved by The Swedish Ethical Review Authority. The patients/participants provided their written informed consent to participate in this study.

## Author Contributions

EM, SL, and MG contributed to the conception and design of the study. MG, L-MA, MB, AL, KN, and J-ÅL provided resources. MB, AL, KN, and J-ÅL established the immunological assays and provided results. EM collected the data and performed data curation. SN contributed to the statistical analysis and EM performed the statistical analysis. All authors contributed to the interpretation of the results. EM and SL wrote the first draft of the manuscript, with MB, KN, and AL writing sections of the manuscript. SL and MG provided supervision. All authors contributed to manuscript editing and revision, and read and approved the submitted version.

## Funding

This work was supported by the Swedish state, under an agreement between the Swedish government and the county councils [ALF agreement ALFGBG-717531 (MG) and ALFGBG-679621 (SL)], by the SciLifeLab/KAW National COVID-19 Research Program [VC-2020-0015 (J-ÅL), KAW-2020-0182 (KN and MG), KAW-2020-0241 (MG); and by the Swedish Heart-Lung Foundation, 2020-0411 (MB)].

## Conflict of Interest

The authors declare that the research was conducted in the absence of any commercial or financial relationships that could be construed as a potential conflict of interest.

## Publisher’s Note

All claims expressed in this article are solely those of the authors and do not necessarily represent those of their affiliated organizations, or those of the publisher, the editors and the reviewers. Any product that may be evaluated in this article, or claim that may be made by its manufacturer, is not guaranteed or endorsed by the publisher.
