# Supplementary material for: Longitudinal Follow Up of Immune Responses to SARS-CoV-2 in Health Care Workers in Sweden With Several Different Commercial IgG-Assays, Measurement of Neutralizing Antibodies and CD4+ T-Cell Responses
Source: Front Immunol. 2021 Nov 2;12:750448. doi: 10.3389/fimmu.2021.750448 (PMC8593002; doi:10.3389/fimmu.2021.750448)
Supplement: Supplementary file 1 [file Image_1.pdf]

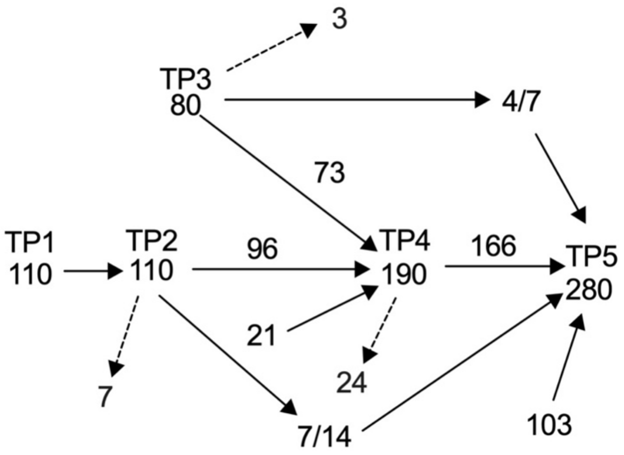

Supplementary Figure 1. The number of health care workers enrolled in the study at the different sampling time points from March 2020 to January 2021. Number of health care workers at each time point (TP), including dropouts (dashed arrows) and additionally included study subjects between all time points.
