# Supplementary material for: Longitudinal Follow Up of Immune Responses to SARS-CoV-2 in Health Care Workers in Sweden With Several Different Commercial IgG-Assays, Measurement of Neutralizing Antibodies and CD4+ T-Cell Responses
Source: Front Immunol. 2021 Nov 2;12:750448. doi: 10.3389/fimmu.2021.750448 (PMC8593002; doi:10.3389/fimmu.2021.750448)
Supplement: Supplementary file 2 [file Image_2.pdf]

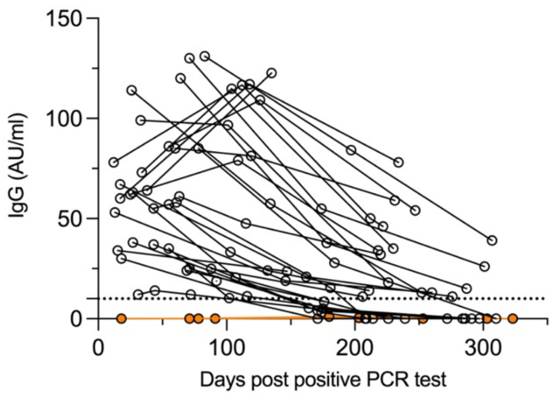

Supplementary Figure 2. Concentrations of IgG, as measured by the N+S-assay, over time in 31 PCR-positive health care workers. Concentrations of IgG (AU/ml) starting from the day of positive PCR test in each study subject. Health care workers with detectable IgG throughout the follow-up to time-point 4 and/or 5 in black (n=29) and without detectable IgG in the assay at any time in orange (n=2).
